# Examining the Dynamic Structure of Daily Internalizing and Externalizing Behavior at Multiple Levels of Analysis

**DOI:** 10.3389/fpsyg.2015.01914

**Published:** 2015-12-17

**Authors:** Aidan G. C. Wright, Adriene M. Beltz, Kathleen M. Gates, Peter C. M. Molenaar, Leonard J. Simms

**Affiliations:** ^1^Personality Processes and Outcomes Laboratory, Department of Psychology, University of Pittsburgh, PittsburghPA, USA; ^2^Human Development and Family Studies, Pennsylvania State University, University ParkPA, USA; ^3^Department of Psychology, University of North Carolina, Chapel HillNC, USA; ^4^Personality, Psychopathology, and Psychometrics Laboratory, Department of Psychology, University at Buffalo, The State University of New York, BuffaloNY, USA

**Keywords:** internalizing, externalizing, personality structure, personality dynamics, psychopathology, multilevel SEM, idiographic modeling, unified SEM

## Abstract

Psychiatric diagnostic covariation suggests that the underlying structure of psychopathology is not one of circumscribed disorders. Quantitative modeling of individual differences in diagnostic patterns has uncovered several broad domains of mental disorder liability, of which the Internalizing and Externalizing spectra have garnered the greatest support. These dimensions have generally been estimated from lifetime or past-year comorbidity patters, which are distal from the covariation of symptoms and maladaptive behavior that ebb and flow in daily life. In this study, structural models are applied to daily diary data (*Median* = 94 days) of maladaptive behaviors collected from a sample (*N* = 101) of individuals diagnosed with personality disorders (PDs). Using multilevel and unified structural equation modeling, between-person, within-person, and person-specific structures were estimated from 16 behaviors that are encompassed by the Internalizing and Externalizing spectra. At the between-person level (i.e., individual differences in average endorsement across days) we found support for a two-factor Internalizing–Externalizing model, which exhibits significant associations with corresponding diagnostic spectra. At the within-person level (i.e., dynamic covariation among daily behavior pooled across individuals) we found support for a more differentiated, four-factor, Negative Affect-Detachment-Hostility-Disinhibition structure. Finally, we demonstrate that the person-specific structures of associations between these four domains are highly idiosyncratic.

## Introduction

Occasioned by patterns of extensive diagnostic co-occurrence, there has been substantial interest in mapping the fundamental nature of psychopathology using quantitative modeling techniques (e.g., Krueger, 1999; Krueger and Markon, 2006; Cramer et al., 2010; Borsboom et al., 2011; Kotov et al., 2011; Wigman et al., 2015). Prime examples of these efforts include the empirically identified *Internalizing* (e.g., unipolar mood disorders, anxiety disorders) and *Externalizing* (e.g., substance use, antisocial behavior) spectra (e.g., Achenbach, 1966; Krueger, 1999; Wright et al., 2013). As has been the case in the basic personality trait literature, research on the structure of mental disorders has prioritized the *between-person* level of analysis (i.e., individual differences). However, there has been increasing interest in studying contextualized dynamic processes associated with psychopathology (e.g., Myin-Germeys et al., 2009; Wichers, 2014). These approaches use a variety of *within-person* data collection and analytic techniques that seek to illuminate the granular and nuanced dynamics of mental disorders. On the surface these two perspectives to understanding psychopathology may seem at odds: one seeking to cast clinical phenomena in terms of generalities, the other pursuing a high degree of specificity. Here we explore bridging these two approaches by examining the structures that emerge from daily diary reports of maladaptive behaviors at the between-person, within-person, and person-specific levels of analysis. In so doing we draw links to efforts in basic personality science that seek to integrate structural and dynamic models by treating traits as ensembles of contextualized processes (e.g., Wright, 2014; DeYoung, 2015; Fleeson and Jayawickreme, 2015; Revelle and Condon, 2015).

### The Structure of Individual Differences in Psychopathology

Psychiatric comorbidity is extensive in the general population (Kessler et al., 1994, 2005), and in clinical samples poly-diagnosis is the rule rather than the exception (Zimmerman and Mattia, 1999). This complicates clinical communication, treatment selection, and frustrates efforts to uncover the pathophysiology, etiology, and maintenance mechanisms of mental illness (Hyman, 2010). As a result, prominent clinical scientists, including the current and past heads of the U.S. National Institute of Mental Health, have called for a complete overhaul of the framework for classifying mental disorders (Hyman, 2010; Insel et al., 2010). Rather than enumerating increasingly detailed categories of disorder, it has been suggested that dimensions of functioning that cut across traditional diagnoses better approximate the structure of psychopathology (e.g., Brown et al., 1998; Widiger and Trull, 2007; Cuthbert and Insel, 2013; Harkness et al., 2014). One promising approach for addressing these issues involves statistically modeling patterns of covariation in diagnosed disorders and symptoms to clarify the natural between-person structure (BP-Structure) of mental disorders (Krueger and Markon, 2006; Wright and Zimmermann, 2015). This approach has been profitably applied to both child (Achenbach, 1966; Lahey et al., 2008) and adult (Krueger, 1999; Krueger and Markon, 2006; Kotov et al., 2011) disorders. In adult psychopathology, as noted above, a well-replicated BP-Structure has emerged based on individual differences in the clustering of disorders and their symptoms into Internalizing and Externalizing spectra (Wolf et al., 1988; Kotov et al., 2010a; Markon, 2010; Wright et al., 2013). This structure has demonstrated strong empirical and statistical evidence for its validity, including invariance across cultures (e.g., Slade and Watson, 2006), gender (Eaton et al., 2012), age groups (Eaton et al., 2011), and time-points within samples (Krueger et al., 1998; Vollebergh et al., 2001).

However, these domains are necessarily broad and decontextualized. In other words, they describe psychopathology in terms of individual differences, not in terms of the within-person or person-specific dynamic processes that often define mental disorders. Indeed, BP-Structural analyses of mental disorder covariation have largely relied on lifetime diagnoses (Krueger, 1999; Kotov et al., 2010a, 2011; Røysamb et al., 2011; Forbush and Watson, 2013; Wright et al., 2013) or some admixture of lifetime and current diagnoses (e.g., Markon, 2010; Blanco et al., 2013; Wright and Simms, 2015). What can be concluded from these studies is that the identified spectra of psychopathology (e.g., Internalizing, Externalizing) reflect latent dimensions of liability for the recognized mental disorders (Krueger and Markon, 2006; Caspi et al., 2014). That is to say, they reflect population-level risk for developing more specific instantiations of psychopathology during the lifespan. These spectra provide invaluable information about patterns of disorder covariation (i.e., co-morbidity), heritability (Kendler et al., 2011), and even the lack of specificity in responses to treatment (Barlow et al., 2010). Yet by themselves these dimensions lack the ability to provide information about proximal etiologies of clinically significant impairment, processes contributing to symptom exacerbation, or possible maintenance mechanisms.

### Psychopathology as Maladaptive Dynamic Processes

Major theories of psychopathology posit processes of disorder development, exacerbation, and maintenance that play out over diverse time scales and frequently involve an interaction between individuals and the context in which they live their lives (e.g., Beck et al., 1979; Teasdale, 1988; Nolen-Hoeksema, 1991; Linehan, 1993; Benjamin, 2005). Indeed, many of the symptoms that define psychiatric disorders are cue- or context-dependent. For instance, social phobia is characterized by intense anxiety and behavioral avoidance when confronted with social or evaluative situations. The hallmark interpersonal impairments of borderline PD are responses to perceptions of significant others’ behavior. The binge-purge cycles of the patient diagnosed with bulimia nervosa reflect a maladaptive and extreme regulatory cycle (e.g., binges and purges both occur in response to heightened negative affect in a specific sequence). Even the blunted hedonic response in depression can be understood as a lack of the normative shift in affect in response to pleasurable events. This has led many researchers to begin studying the dynamic processes of psychopathology as they unfold in the naturalistic settings of daily life (e.g., Shiffman et al., 2002; Wegner et al., 2002; Silk et al., 2003; Ebner-Priemer et al., 2007; Trull et al., 2008; Sadikaj et al., 2013; Pe et al., 2015; see also Myin-Germeys et al., 2009 for a review).

This approach has provided much needed systematic empirical confirmation of the clinical description of psychiatric phenomena (e.g., affective instability in borderline PD; Russell et al., 2007; Trull et al., 2008) and has offered new insights into maladaptive behavioral sequences (e.g., individuals diagnosed with borderline PD are more likely to respond to perceived quarrelsomeness with negative affect, but no more likely to respond to negative affect with quarrelsomeness than controls; Sadikaj et al., 2013). Interestingly, as debates about psychiatric nosology have been pushing the field away from disorder-specific symptoms and toward dimensions that cut across traditional diagnoses, the study of dynamic processes in psychopathology has instead been emphasizing highly specific micro-processes (e.g., Wichers, 2014; Fried, 2015). It is notable that the majority, but not all, of the research studying dynamic processes in naturalistic settings have used a diagnostic group based design (e.g., comparing patients vs. community controls). Although there are plenty of good reasons for selecting circumscribed diagnostic groups for study (e.g., ensuring sufficient levels of pathology; maximizing statistical power in very expensive and difficult to collect data), this approach is at odds with efforts to collapse across categories to study dimensions of shared impairments (Krueger and Markon, 2006; Insel et al., 2010).

Thus, there is a tension between different areas of clinical science, which presumably share the same goal of clarifying the nature of psychopathology. The tension created is one between emphases on BP-Structure and within-person processes, which is certainly not a novel challenge (cf. Titchener, 1898). Taking as a given that both empirical thrusts have important information to contribute, the question becomes how best to integrate advances in the between-person structure of individual differences with the within-person study of dynamic processes (Wright, 2011; Hopwood et al., 2015).

### Conceptually Integrating Between-Person Structure and Within-Person Dynamic Processes

A model for resolving the tension between investigations that focus on BP-Structure and within-person dynamic processes can be found in contemporary personality theory. Akin to the quantitative modeling of covariation in mental disorders, personality researchers invested heavily in the modeling of dispositional attributes that ultimately resulted in the Big-Five/Five-Factor Model of personality (for reviews see Digman, 1990, 1996; Goldberg, 1993; Wright, in press). Paralleling these investigations, researchers interested in personality processes have sought to study the within-person temporal dynamics of specific thoughts, feelings, and behavior, which are the behavioral building blocks of personality traits (e.g., Carver and Scheier, 1982; Larsen, 1987; Mischel and Shoda, 1995; Eid and Diener, 1999; Fleeson, 2001; Moskowitz and Zuroff, 2004; Cervone, 2005). Until recently and with few exceptions (e.g., Borkenau and Ostendorf, 1998), studies of the BP-Structure and dynamic processes of personality have largely proceeded separately (Read et al., 2010). There is now increasing interest in meaningful synthesis of models of individual differences in structure and the putative underlying dynamic processes that give rise to this structure (e.g., Fleeson, 2007; Fleeson and Gallagher, 2009; Fournier et al., 2009; DeYoung, 2015; Fleeson and Jayawickreme, 2015; Revelle and Condon, 2015). At the risk of oversimplifying, these integrative approaches take the domains outlined by BP-Structural models of individual differences (e.g., the Big-5), and use them as the orienting dimensions to organize hypotheses and investigations into the patterning of within-person dynamic processes (e.g., McCabe and Fleeson, 2012; Wright et al., 2015).

This integrative approach may be viable in psychopathology research given that the structures of personality and psychopathology are meaningfully overlapping (Wright and Simms, 2015). Long hypothesized, going back to antiquity and the writings of Hippocrates and Galen, evidence for the link between personality/temperament and mental disorders has is now quite robust. For one, several meta-analyses show that personality trait ratings and mental disorder diagnoses are strongly associated (e.g., Saulsman and Page, 2004; Ruiz et al., 2008; Samuel and Widiger, 2008; Kotov et al., 2010b). The meta-analytic results show that disorders falling within the Internalizing spectrum demonstrate strong associations with Neuroticism and Detachment (i.e., Introversion), whereas disorders falling within the Externalizing spectrum are most strongly associated with Disinhibition (i.e., low Conscientiousness and Impulsivity) and Antagonism (i.e., low Agreeableness).

Moreover, the hierarchical organization of personality traits and mental disorders bear unmistakable resemblance. A consistent finding is that at the level of two higher-order domains, dimensions of maladaptive personality bear close resemblance to the Internalizing and Externalizing spectra (Markon et al., 2005; Kushner et al., 2011; Wright et al., 2012; Wright and Simms, 2014). In these models, the Internalizing domain subsumes lower-order domains of Negative Affectivity and Detachment, and the Externalizing domain subsumes Disinhibition and Antagonism. Further, there is now accumulating evidence from models that incorporate broader sampling of psychopathology for additional spectra labeled Antagonism and Detachment/Anhedonic or Pathological Introversion (Markon, 2010; Kotov et al., 2011; Røysamb et al., 2011; Wright and Simms, 2015). Although direct evidence from hierarchical structural models of DSM diagnoses is lacking, the conceptual convergence with hierarchical models of personality suggests that a disorder based Antagonism domain can be joined with traditional indicators of disinhibitory pathology to form a broader Externalizing factor (e.g., Krueger et al., 2007), whereas Pathological Introversion would join affective disorders to define a higher order domain of Internalizing. Thus it is expected that with further targeted research the hierarchy of mental psychopathology and personality will largely converge.

Taken together, this suggests that much like contemporary personality science, investigations into within-person temporal processes of mental disorders could benefit from using the same empirically derived domains (e.g., Internalizing, Externalizing) that organize between-person differences in psychopathology in traditional cross-sectional research. As such, demonstrating similarities in structure at both levels would be the minimum requirement to ensure success of this approach. However, it is unknown whether the within-person structure (WP-Structure) that emerges from the temporal patterning of specific behaviors over time mirrors the BP-Structure of individual differences in the expression of those same maladaptive behaviors either at the higher-order level of Internalizing and Externalizing or possibly with lower-order differentiation of sub-factors within each domain.

### Methodological Integration of Structure and Dynamic Processes

Joining models of between-person individual differences with the study of within-person dynamic processes immediately raises the issue of how to appropriately model and test whether such a marriage will succeed. Specifically, it involves modeling data that has a multilevel structure, with many time-points or occasions of measurement nested within individuals. There are two sources of variance in this type of data: variability associated with between-person differences in mean item endorsement, and variability associated with within-person, time-point specific deflections around those means. As Molenaar (2004) has shown, the structure of within-person covariation of behaviors is mathematically distinguishable from the covariation patterns of between-person differences in the mean levels of these behaviors (see also Nesselroade and Molenaar, 1999; Borsboom et al., 2003; von Eye and Bergman, 2003; Grice, 2004). That is to say, there is no guarantee that the same structure holds at both levels. Furthermore, the same WP-Structure may not apply to all individuals (Molenaar, 2004; Molenaar and Campbell, 2009). Indeed, for many applications, it is the person-specific (i.e., idiographic) structure (PS-Structure) that is of greatest interest. For instance, when it comes to tailoring and applying a behavioral intervention, substantial individual heterogeneity compels the development of a “model of the individual.”

A note on terminology is warranted. Here we draw a distinction between three tiers of structural analysis that are available when modeling intensive longitudinal data. BP-Structure refers to traditional conceptions of cross-sectional individual differences, and is derived from the covariation of behaviors averaged across time-points. Thus, it is time-invariant, or *static* in nature. We additionally consider two levels of *dynamic* structure. For the first dynamic approach, we use the term “WP-Structure” for the structure of temporal covariation in behaviors, pooled in whole or in part across individuals as is common in multilevel analysis. In other words, it is the within-person, dynamic patterning of behaviors, controlling for average levels, but shared, at least in part, across all individuals in the sample. For the second approach we use the term “PS-Structure” for person-specific models of temporal covariation that are based solely on a single subject’s multivariate time-series.

Several approaches have been developed for the appropriate structural analysis of intensive longitudinal data in groups of individuals. Multilevel structural equation modeling (MSEM; Muthén, 1991, 1994) generally offers a top–down approach, decomposing the total variance of the observed variables into the latent between- and within-person portions, and then fitting a model to each. It can be considered a top–down method because in MSEM a WP-Structure is specified that is then fitted to all individuals simultaneously (see also Shumway and Stoffer (2006) section 6.11 for a time-series perspective on this approach). Other methods adopt a bottom–up approach, starting with the structure of individuals and finding communalities in the individual data structures [e.g., the Integrated Trait-State Model (Hamaker et al., 2007) or Multilevel Simultaneous Component Analysis (Timmerman, 2006)], or iteratively fitting group- and individual-specific SEMs [e.g., Group Iterative Multiple Model Estimation (Gates and Molenaar, 2012)]. These approaches arrive at partially shared structure or parameters.

Methods for deriving PS-Structures involve the idiographic analysis of a single individual’s multivariate time-series. Certain methods are mathematically and conceptually parallel to the analysis of multivariate structure across individuals [e.g., P-technique Factor Analysis (Baldwin, 1946; Cattell, 1966)] or augment the analysis with temporal information by including a block-Toeplitz matrix [Dynamic Factor Analysis (Molenaar, 1985)] or using a multiple indicator vector autoregression moving average model (Hamaker et al., 2005). Recent developments include unified SEM (uSEM), which combines vector autoregression with SEM (Kim et al., 2007). Although similar, these methods differ in their emphasis on latent variables and inclusion of temporal lags [i.e., modeling associations from one time point (*t*-1) to the next (*t*)].

All of these methods share the ability to appropriately handle multilevel data structures, and each offers distinct advantages and disadvantages that need to be weighed with the specific modeling demands of the research question. To highlight a key distinction, the models of dynamic structure (i.e., WP-Structure or PS-Structure) differ in their level of complexity and flexibility in allowing for differences in structure across individuals. The least complex is the shared WP-Structure derived from MSEM with the most being the PS-Structure derived from idiographic analyses. Arguably, PS-Structure offers the most precise match to any given individual’s actual patterning of behavior. At the same time, when investigating large samples of individuals there may be value in using a more constrained approach like MSEM to appropriately reduce highly dimensional data into coherent but manageable factors. Practically speaking, investigators need to balance traditional assessment considerations (e.g., reliability of estimates, measurement error, bandwidth fidelity tradeoffs, etc.) with modeling complex nuanced dynamic processes.

### The Current Study

The overarching goal of the current study was to provide a bridge between research paradigms that adopt divergent approaches to clarifying the fundamental nature of psychopathology. More precisely, we sought to provide a much needed conceptual link between work that has established transdiagnostic domains or crosscutting dimensions of psychopathology in cross-sectional data, and more recent efforts to understand the complex dynamic processes that characterize mental disorders as they play out in daily life. Toward this aim, we posed the following specific questions. First, *does the BP-Structure of individual differences in daily endorsement of maladaptive behaviors conform to the latent structure of psychiatric diagnoses* (i.e., Internalizing and Externalizing)? Second, *can the same structure be applied to the WP-Structure of dynamic daily fluctuations in maladaptive behaviors*? Third, *is there PS-Structure heterogeneity in the daily and cross-day (i.e., lagged) links among the dimensions identified by WP-Structural analyses*?

To answer the first two questions we estimated multilevel confirmatory factor analyses (i.e., MSEM) in a sample of individuals diagnosed with PDs who completed daily diaries of maladaptive behaviors over 100 consecutive days. We tested two *a priori* models for both the BP- and WP-Structures. We based our tested models on research on the overlap in BP-Structures of personality and psychopathology discussed above. Thus, we tested two-factor Internalizing and Externalizing models, as well as a nested four-factor model that partitioned Internalizing into its lower order domains of Negative Affectivity and Detachment, and partitioned Externalizing into lower order domains of Disinhibition and Hostility (representative of the broader Antagonism domain). Finally, we then addressed the third question by using uSEM to examine the person-specific interplay among components of this dynamic structure for a subset of participants, mapping networks in which Internalizing and Externalizing sub-factors influence each other at multiple temporal lags.

## Materials and Methods

### Participants

The sample used in this study was collected as part of a project designed to investigate general daily processes of behavior in individuals with PD. As such, recruitment targeted individuals diagnosed with *any* PD. Participants were recruited from a clinical sample (*N* = 628) enrolled in an ongoing study to improve efficient measurement of PD (Simms et al., 2011, under review). Participants were recruited into the broader clinical sample by distributing flyers at mental health clinics across Western New York, and were eligible for participation in the parent study if they reported psychiatric treatment within the past 2 years. Participants received structured clinical interviews by trained assessors for clinical syndromes and PDs using the sixth edition of the Mini International Neuropsychiatric Inventory (MINI; Sheehan and Lecrubier, 2010) and a version of the Structured Clinical Interview for DSM-IV-TR PDs (SCID-II; First et al., 1997), respectively. Only specific PD diagnoses were evaluated; PD-NOS was not evaluated or diagnosed. Disorder-level Kappas from independent ratings of a subset of participants (*n* = 120) were strong (*Mdn K* = 0.96; *range* = 0.66–1.00). Those who met the threshold for *any* PD diagnosis on the clinical interview were contacted for possible participation in the current daily diary study. The sole additional requirement for participation was daily Internet access via computer or mobile device.

One hundred and sixteen participants attended the baseline assessment for the daily diary study. Due to the focus on variability in behavior in this study, only participants providing at least 30 days worth of data were included to ensure reliable estimates of variability. Only 15 individuals were excluded for providing less than 30 diaries, resulting in an effective sample size of 101. Of these participants, 66 (65.3%) were female, and the majority reported being either White (82.2%) or African American (14.9%). On average, time between diagnostic interview and the initial assessment in this study was 1.4 years (*Range* = 1.2–1.7 years; *SD* = 0.16 years). The rates of PD diagnoses were as follows: 35.6% paranoid, 13.9% schizoid, 16.8% schizotypal, 7.9% antisocial, 36.6% borderline, 2.0% histrionic, 19.8% narcissistic, 53.5% avoidant, 5.9% dependent, 50.5% obsessive-compulsive. The average number of PD diagnoses per participant was 2.4. Additionally, 62.4% were diagnosed with mood disorders, 69.3% with anxiety disorders, 8.9% with psychotic disorders, and 23% with substance/alcohol use disorders. Demographics for the retained sample are presented in **Table 1**. Relative to the pool of 628 participants the current sample was drawn from, no differences were found on Age, Sex, or Employment Status. We found differences on Race (φ = 0.19), Marital Status (φ = 0.13), Educational Attainment (φ = 0.16), and Income (φ = 0.17), all of which were of small effect. Participants in the retained sample were less likely to be Black, and were more likely to be in higher Income or Educational Attainment categories. The retained sample was more likely to be married and less likely to be divorced or separated. Seventy-two percent of participants reported current mental health care treatment, 14% within the last year, and the remainder longer than 1 year prior to the daily diary protocol.

**Table 1:** Sample demographics.

	*N/M*	%/*SD*
Age	44.9	13.3
**Gender**		
Male	35	34.7
Female	66	65.3
**Race/Ethnicity**		
White	83	82.2
Black	15	14.9
Native American	3	3.0
Hispanic	5	5.0
**Education**		
No high school diploma	6	6.0
High school diploma	16	15.8
Some college	34	33.7
College degree	28	27.7
Graduate/Professional	17	16.8
**Employment**		
Employed	35	34.7
Unemployed	13	12.9
Disabled	33	32.7
Retired	9	8.9
Student	5	5.0
Homemaker	3	3.0
**Income**		
Less than $15,000	26	25.7
$15,000–$29,999	23	22.8
$30,000–$44,999	20	19.8
$45,000–$59,999	13	12.9
More than $60,000	19	18.9
**Marital Status**		
Married	27	26.7
Widowed	5	5.0
Divorced	18	17.8
Separated	3	3.0
Never Married	48	47.5

*N = 101.*

### Procedure

A complete description of the study was provided, and written informed consent was obtained prior to participation in accordance with the Declaration of Helsinki. The University at Buffalo institutional review board approved all study procedures. Participants attended an initial in-person training and assessment session during which study procedures were explained, and a battery of self-report measures was completed via computer. Starting the evening of the in-person assessment, participants were asked to complete daily diaries assessing daily interpersonal behavior, affect, symptoms, stress, and functioning via secure website every evening for 100 consecutive days. Surveys were to be completed at roughly the same time each day, between 8 pm and 12 am. However, participants were allowed to deviate from this schedule if necessary (e.g., working nightshift) so long as (a) they completed diaries at the end of their day, and (b) the diaries were completed at roughly the same time each day. Participants received daily email reminders and also were provided several paper diaries they could use in the event of technological difficulties. Compliance rates were very high, with a total of 9,041 diaries completed by participants in this study after data cleaning (*Mdn* = 94 days, *M* = 89.5 days, *range* = 33–101 days, 90% > 60 days), a small fraction of which were done by paper (∼2% of completed diaries).^1^ Compensation was provided for daily participation at the rate of $100 for ≥80% participation, and prorated at $1/day for <80%. Participation also was incentivized though recurring raﬄes ($10 drawing every 5 days for those providing at least four diaries) and drawings for additional money and tablet computers at the end of the study, with the odds of winning proportionally tied to participation.

### Measures

**FIGURE 1:**
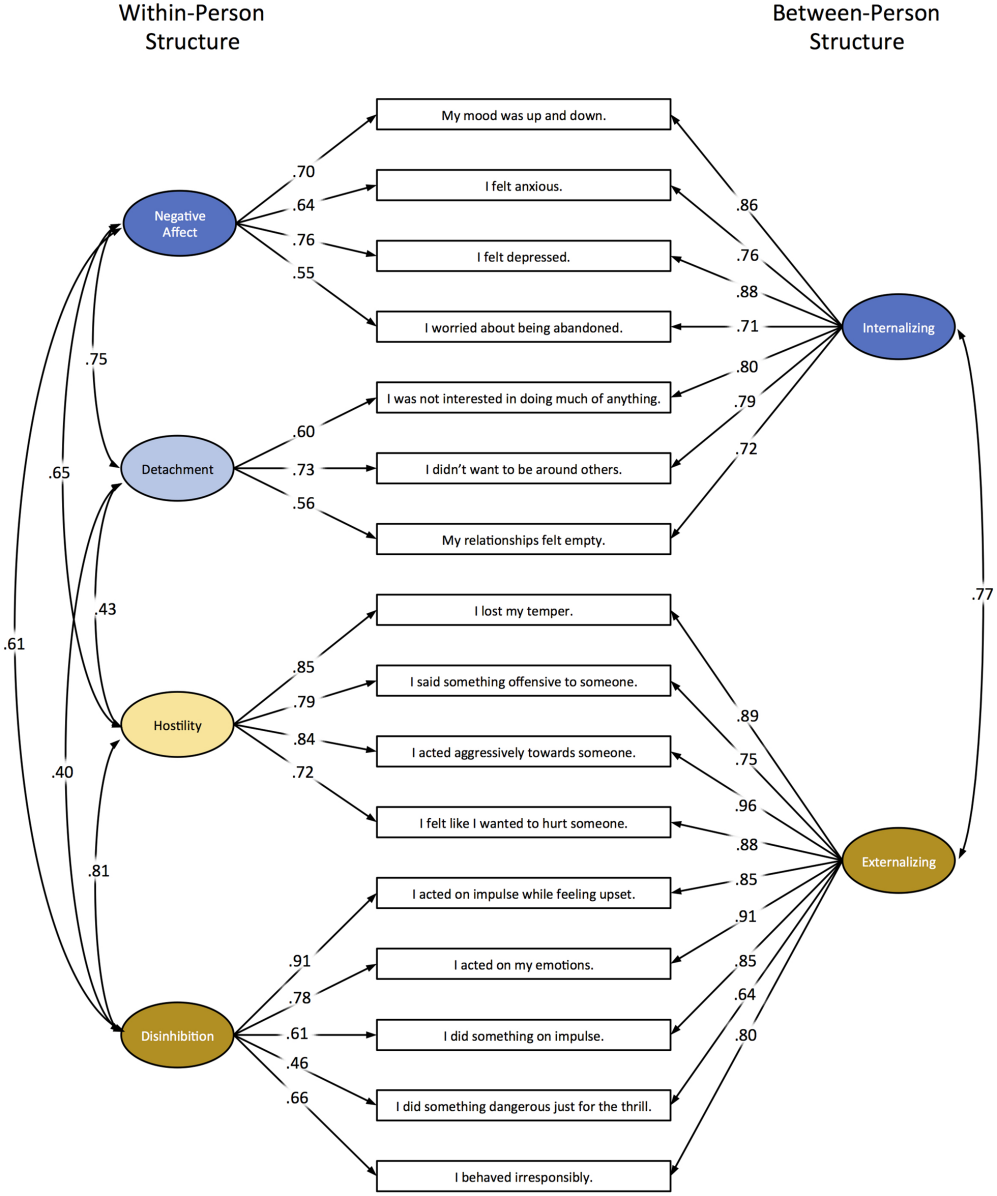
**Diagram of multilevel confirmatory factor analysis model with standardized factor loadings and latent covariances.** Item specific residual variances and factor variances not depicted.

Daily behaviors were measured using 16 items created for the purpose of this project. The specific questions used in this study are listed in the boxes denoting observed variables in **Figure 1**. These 16 items were selected for their relevance to the current study from a larger set of behaviors designed to provide broad coverage of the daily manifestations of personality pathology. Items were intended to reflect concrete behavioral manifestations of broad domains of personality pathology as they might occur in daily life. Items were written so that they were not so extreme as to have problematically low endorsement on a daily basis, and participants were given an 8-point response scale for each item anchored with *Not at All* (0) and *Very Much So* (7). Prior work in this sample has examined the basic descriptive features of these and the additional excluded items, including rates of endorsement, levels of (in)stability, and associations with the DSM-5 personality trait domains (Wright and Simms, under review). Of the 16 items used in this study, four were hypothesized primarily to reflect Negative Affectivity (multilevel coefficient alphas using Geldhof et al., 2014 approach were α_Between_ = 0.88, α_Within_ = 0.76), three primarily to reflect Detachment (α_Between_ = 0.84, α_Within_ = 0.64), four primarily to reflect Hostility (α_Between_ = 0.93, α_Within_ = 0.87), and the remaining five to primarily reflect Disinhibition (α_Between_ = 0.92, α_Within_ = 0.82).

Additionally, symptom counts from diagnostic interviews for major depressive disorder, dysthymia, generalized anxiety disorder, social phobia, post-traumatic stress disorder, alcohol use disorder, substance use disorder, child conduct, antisocial, avoidant, dependent, borderline, narcissistic, histrionic, and paranoid PDs were used to develop a interview based structural model described below. Reliabilities (K’s) are reported above.

### Data Analysis

As described in the introduction, our analyses incorporate both MSEM and uSEM, and we describe each in turn.

#### Testing BP- and WP-Structure of Maladaptive Daily Behaviors

The first two questions we sought to answer concerned whether the structure derived from individual differences in psychiatric diagnoses could be adequately fit to the daily diary data at the between- and within-person levels. We additionally sought to use the WP-Structure to reduce the dimensionality and complexity of the data for subsequent idiographic analysis. Therefore we selected MSEM as an analytic framework. MSEM extends traditional multilevel regression (i.e., hierarchical linear modeling; random coefficient regression) to multilevel covariance and mean structural modeling (Muthén, 1991, 1994). It does so by partitioning the total variance in the observed variables into the latent between-person variance (commonly referred between-cluster or between-group variance), and the observed within-person (also within-cluster or within-group) variance (Muthén, 1991). The partitioned variance can then be used to calculate both between- and within-person covariance matrices. Although the within-person covariance matrix is straightforwardly calculated and understood, calculation of the between-person covariance matrix is more complex (e.g., it is weighted for differences in cluster size) and is conceptually akin to the covariance among random intercepts (see Muthén, 1994 and Heck, 1999 for technical details, and Reise et al., 2005 and Preacher et al., 2010 for accessible summaries).

With the variance thus partitioned, MSEM offers the opportunity to separately estimate and compare between- and within-person structures by fitting standard latent variable models, like confirmatory factor analysis (CFA). A multilevel CFA was employed here and allows for potentially different factor structures to emerge at each level of the data. In the current context, the between-person structure reflects the pattern of covariation in average item endorsements over the course of the study, or, conceptually, the trait structure of these behaviors. In contrast, the within-person structure reflects the tendency for individual behaviors to covary at the daily level, or, conceptually, the dynamic structure of these behaviors. Here we estimate a series of between- and within-person factor models to determine the optimal structure of daily maladaptive behaviors sampled in this study.

For our first aim, we primarily were interested in testing whether a two-factor (Internalizing, Externalizing) model would acceptably fit the data, and if so, whether a four-factor model (Negative Affect, Detachment, Hostility, Disinhibition) improved upon this fit at the BP and WP levels. In addition to the 2- and 4-factor models of interest, we estimated one-factor models as a point of comparison. To test this, we estimated a series of MSEM models in Mplus version 7.31 (Muthén and Muthén, 1998–2012). Due to significant skew and kurtosis in the Externalizing behavior items, we treated all items as ordinal, and estimated multilevel CFAs using a robust (mean adjusted) weighted least squares approach (WLSM) on the polychoric correlation matrix. Model fit testing in MSEM can be challenging because the χ^2^ test and alternative fit indices are derived from the comparison of the observed and implied covariance for both the between- and within-person matrices simultaneously. Therefore it is difficult to disentangle sources of ill model fit across levels. To address this complication, we adopted Ryu and West’s (2009) approach, which eliminates any source of ill fit from a given level by fitting a saturated model (i.e., zero *df*), while models of interest are tested in the other level. For example, first a saturated model was fit on the within-person level, and hypothesized models were fit to the between level, and then this process was reversed, fitting a fully saturated model at the between level, and estimating models of interest at the within-person level. Saturated models fit the data perfectly, and therefore they do not contribute to lack of fit, so any source of ill fit comes from the models at other levels. Ryu and West (2009) provide additional details about appropriate calculation of alternative fit indices for independence models at each level. Although evaluation of global model fit in MSEM remains an understudied topic, we considered the χ^2^ test, as well as several alternative fit indices, using their single-level SEM recommended cutoffs (Hu and Bentler, 1999). These include the root mean square error of approximation (RMSEA) with values <0.05 for good model fit, comparative fit index (CFI) with values near or >0.95 indicative of good model fit, and the SRMR, with values <0.08 indicative of good model fit. Because we used the WLSM estimator, nested models were compared using the strictly positive Satorra–Bentler scaled χ^2^ difference test (Satorra and Bentler, 2010).

In order to test the validity of our retained model, in a final MSEM we estimated interview-based Internalizing and Externalizing spectra using data from the structured clinical interview that were administered on initial assessment, and used these as predictors of the between-person factors from the daily behaviors. We used Kotov et al. (2011) structure, which includes PD diagnoses in the model, as a template to select relevant variables for our interview based model. We combined the diagnoses from Kotov et al.’s (2011) Externalizing and Antagonism domains in order to arrive at a broader Externalizing domain that would better match out daily behaviors. Thus, our interview-based Internalizing model was indicated by symptom counts for major depression, dysthymia, social phobia, post-traumatic stress, generalized anxiety, avoidant, dependent, and borderline PDs. The interview-based Externalizing factor was indicated by symptom counts for alcohol use, drug use, childhood conduct, adult antisocial, narcissistic, histrionic, paranoid, and borderline PDs (Please see Appendix A in Supplementary Material for example MSEM syntax).

#### Exploring Person-Specific Structures of Maladaptive Daily Behavior

Our second aim was to demonstrate how the optimal WP-Structure derived from the MSEM could be leveraged to inform person-oriented personality processes, providing a picture of the data at a third conceptual level of analysis. To accomplish this, we implemented individual-level unified structural equation modeling (uSEM; Kim et al., 2007; Gates et al., 2010). This approach combines SEM and vector autoregression of a single participant’s daily diary data in order to map the interplay among personality factors, that is, how variability in each factor is influenced by the contemporaneous (occurring on the same day, *t*) and lagged (occurring on previous days, *t*-1) variability in other factors. The model with a mean fixed zero is defined as:

$$\eta (\text{t})=A\eta (\text{t})+\Phi_{1}\eta (\text{t}-1)+\Phi_{2}\eta (\text{t}-2)+\ldots +\Phi_{\text{j}}\eta (\text{t}-\text{j})+\zeta (\text{t}),$$

where η(t) is the *p*-variate time series to be explained at day *t* = 1, 2,…, T, with p the number of MSEM-derived within-person factors and T the number of daily diary entries, A the (p,p)-dimensional matrix of contemporaneous regression coefficients explaining how each factor is influenced by other factors on the same day, Φ_q_ is the (p,p)-dimensional matrices of regression coefficients at lag *q* = 1, 2,…, j explaining how each factor is influenced by itself or other factors from previous days, and ζ is the *p*-variate error process, lacking sequential dependencies and having a zero mean and a diagonal contemporaneous covariance matrix. Simulation studies have found that incorporating contemporaneous and lagged effects simultaneously greatly improves reliability of results when compared to models that solely include one type of effect (Gates et al., 2010) and has been successfully applied to neuroimaging and observational data (Hillary et al., 2011; Beltz et al., 2013), with the present study being the first application to daily dairy data.

We fit uSEMs to the daily diary data of four exemplar participants in a data-driven fashion (cf. Gates et al., 2010) that accounted for the presence of multiple solutions (a characteristic of SEMs; MacCallum et al., 1993), and that satisfied the assumption of independent errors. We used LISREL for the analyses (Jöreskog and Sörbom, 1992). Model fitting was conducted in several steps. First, the items contributing to each MSEM within-person factor were averaged to create a factor composite score, as is commonly done in individual differences research. Second, a null uSEM model (i.e., no contemporaneous or lagged effects estimated) of the first order was fit to the data using the block Toeplitz method (cf. Molenaar, 1985). Third, Lagrange Multiplier tests (i.e., modification indices; Sörbom, 1989) were used to free and estimate the parameter in the A or Φ_1_ matrix that would most improve model fit; this process iterated until no parameter would significantly (at *p* ≤ 0.05) improve model fit it if were freed. Multiple solutions could occur during this iterative process if modification indices showed that two parameters would equally improve model fit (i.e., their Lagrange Multiplier tests were equivalent). In these cases, each parameter was freed and estimated in a separate solution, and the iterative estimation process continued independently for each (with the possibility of further separations), generating a set of possible solutions (cf. Beltz and Molenaar, in revision). Fourth, non-significant parameters were trimmed from the models. Fifth, model fit was evaluated for the solutions using alternative fit indices, with two of the following four required to indicate excellent fit (Brown, 2006): RMSEA ≤0.05, SRMR ≤ 0.05, CFI ≥ 0.95, NNFI ≥ 0.95. Sixth, if multiple solutions occurred during the model fitting process, then the optimal solution was selected by choosing the model with the lowest AIC, a selection criterion employed in previous work (Akaike, 1974; MacCallum et al., 1993; Beltz and Molenaar, in revision). Seventh, the solution was examined for independent residuals using a posteriori model validation (Box and Jenkins, 1970). Specifically, one-step-ahead prediction errors were generated from the model and tested for white noise (cf. Beltz and Molenaar, 2015). If white noise was found, then a first order uSEM was appropriate for the data, and the solution was accepted. If white noise was not found, then a first order solution was insufficient for capturing all sequential dependencies in the data, and steps two through seven were repeated for a second order uSEM (i.e., a model with A, Φ_1_, and Φ_2_ matrices; Please see Appendix B in Supplementary Material for example uSEM synt).

## Results

Global model fit and model fit comparisons for the MSEM analyses can be found in **Table 2**. Starting with the between-person level, all estimated models were considered a good fit to the data using the chi-square tests, which were uniformly non-significant. This was expected due to the low-powered test with a between-person sample size of 101. The RMSEAs and CFIs also were excellent, although the SRMR was only acceptable in models with 2 and 4 factors. The likelihood ratio test indicated that model fit improved going from 1 to 2 factors, but a four-factor model did not significantly improve the fit. As such, we selected a two-factor structure as the optimal BP model in these data. For the WP level, the chi-square tests were uniformly significant. This was expected due to the high-powered test with 9,041 within-person observations. The RMSEA and CFI suggested all models were good fitting, although each improved appreciably going from 1 to 4 factors. The SRMR was only acceptable in models with 2 and 4 factors. Finally, the chi-square difference test strongly favored a four-factor solution. Thus, our final retained model differed in structure across levels of analysis, with two factors at the between-person level, and 4 factors at the within-person level of analysis.^2^ The model with standardized parameter estimates can be found in **Figure 1**.

**Table 2:** Multilevel confirmatory factor analysis model fit and model fit comparisons.

	Model fit							Model comparisons			
Model	*df*	χ^2^	χ^2^*p*	RMSEA	CFI	SRMR_W_	SRMR_B_	Models	Δχ^2^*df*	Δχ^2^_SB_	Δχ^2^_SB_*p*
**Between**								
(1) SW/1B	104	52.07	1.00	0.000	1.00	–	0.090	–	–	–	–
**(2) SW/2B**	**103**	**21.16**	1.00	**0.000**	**1.00**	–	**0.060**	**1 vs. 2**	**1**	**30.58**	**<0.001**
(3) SW/4B	98	16.63	1.00	0.000	1.00	–	0.055	2 vs. 3	5	4.78	0.443
**Within**								
(4) 1W/SB	104	2860.36	<0.001	0.054	0.95	0.111	–	–	–	–	–
(5) 2W/SB	103	1284.23	<0.001	0.036	0.98	0.062	–	4 vs. 5	1	1581.46	<0.001
**(6) 4W/SB**	**98**	**478.76**	**<0.001**	**0.021**	**0.99**	**0.057**	**–**	**5 vs. 6**	**5**	**794.87**	**<0.001**

*Selected model at each level bolded. Models were estimated treating all observed variables as categorical using mean adjusted weighted least squares (WLSM in MPlus) as the estimator. In the Model column the numerals reflect number of factors estimated, and W, within; B, between; S, Saturated. RMSEA, root mean square error of approximation; CFI, Comparative fit index; SRMR, standardized root mean square residual; Δχ^*2*^_*SB*_, Satorra-Bentler chi-square difference test.*

**FIGURE 2:**
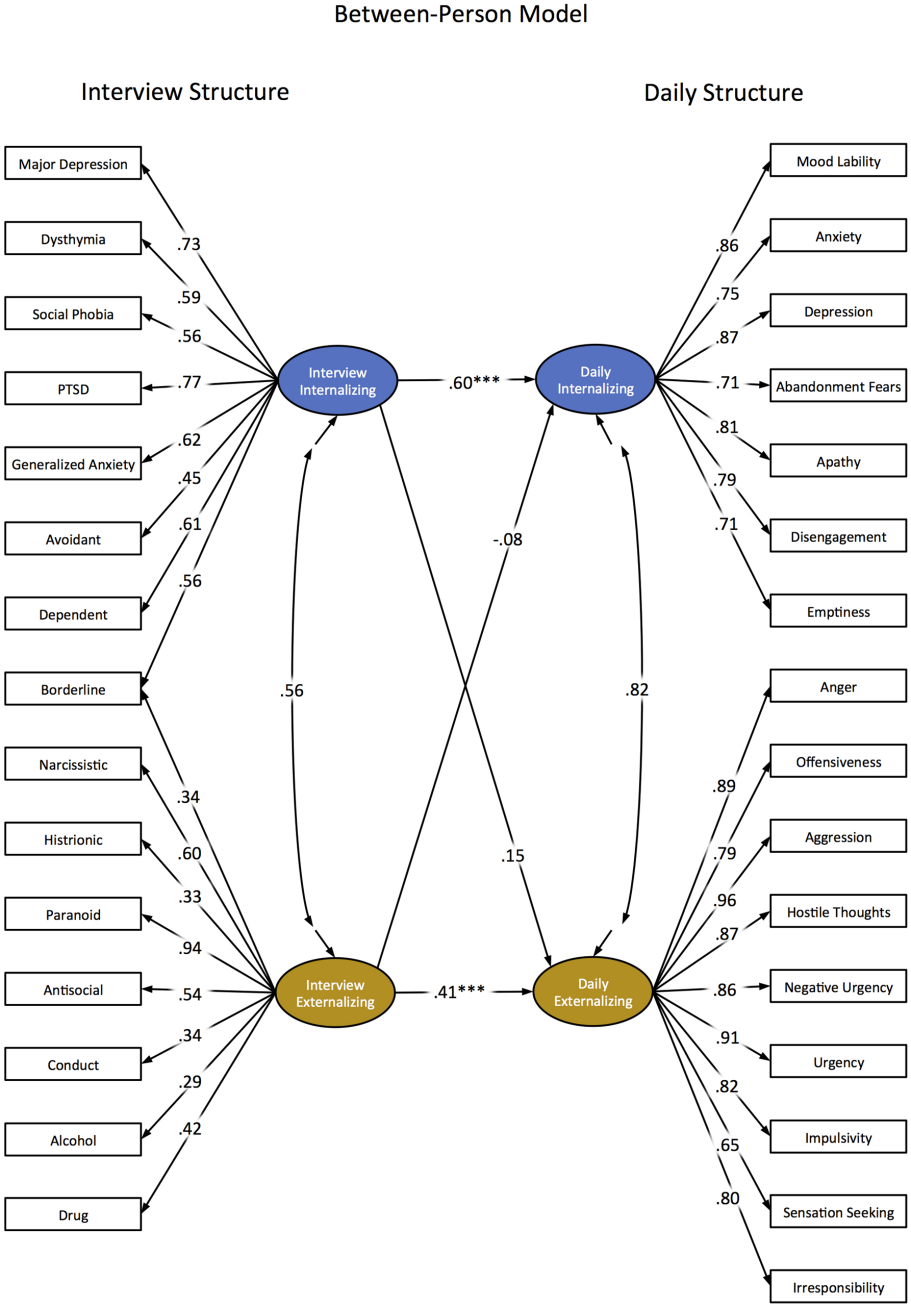
**Diagram of multilevel structural equation model (standardized estimates) with interview based Internalizing and Externalizing factors predicting daily diary based Internalizing and Externalizing Spectra.** Item specific residual variances and factor variances not depicted. Asterisks depict significant regression paths between latent factors (^∗∗∗^*p* < 0.001), but significance is not provided for other parameters.

To test the validity of this model, we estimated Internalizing and Externalizing factors from the original clinical interviews and regressed the daily diary based Internalizing and Externalizing factors on each of these. This resulted in an excellently fitting model [χ^2^_(525)_ = 568.54, *p* = 0.09; RMSEA = 0.003; CFI = 1.00; NNFI = 1.00; SRMR_Within_ = 0.05, SRMR_Between_ = 0.09]. Relevant model parameter estimates can be found in **Figure 2**. We found that the interview-based Internalizing factor was a significant predictor of daily Internalizing (β = 0.60; 95% confidence interval = 0.38 to 0.81; *p* < 0.001), but not of daily Externalizing (β = 0.15; 95% confidence interval = -0.09 to 0.38; *p* < 0.22). In contrast, we found that the interview-based Externalizing factor was a significant predictor of daily Externalizing (β = .41; 95% confidence interval = 0.18 to 0.63; *p* < 0.001), but not of daily Internalizing (β = -0.08; 95% confidence interval = -0.32 to 0.16; *p* < 0.55). Thus, our between-person factors estimated from daily diaries evidence significant associations with corresponding traditional interview based factors, and these associations were specific.

**FIGURE 3:**
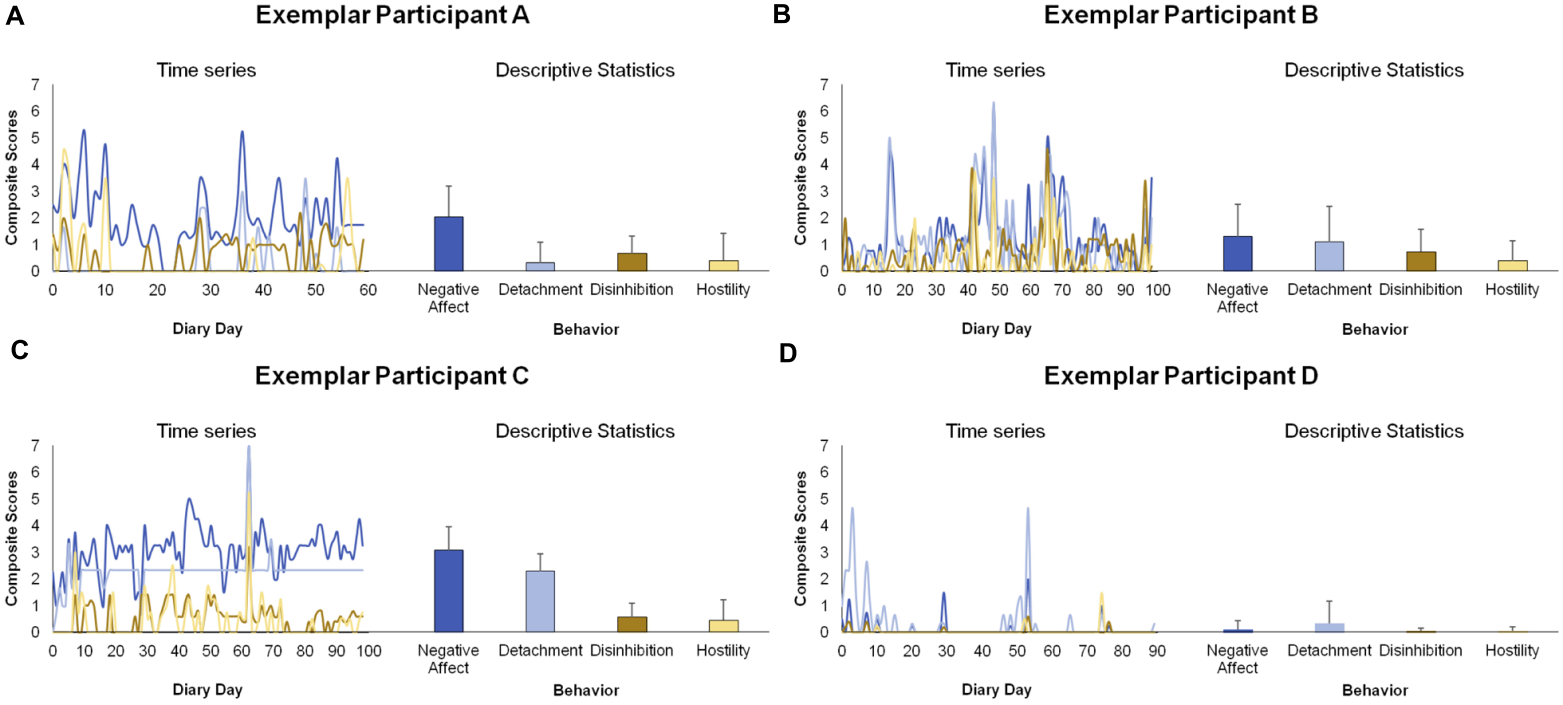
**Time series plots and descriptive statistics for composite scores of the four within-person factors used in person-oriented uSEM analyses for each of four exemplar participants **(A–D)**.** Time series plots show the composite scores for each daily diary. Bar graphs show the means and standard deviations of the scores across all diaries. Within-person factors in shades of blue define the between-person factor of internalizing, and within-person factors in shades of yellow define the between-person factor of externalizing.

Next, we examined person-oriented personality processes by mapping with uSEM the interplay among the four within-person factors for four exemplar participants; four, three, five, and four items as indicated from the MSEM analysis were averaged to create the Negative Affect, Detachment, Disinhibition, and Hostility composite scores, respectively; see **Figure 1**. Time series plots and descriptive statistics for each participant’s scores are shown in **Figure 3**. Notice how some characteristics of participants’ daily responses shown in the time series plots were independent of the descriptive statistics (i.e., means and standard deviations shown in the bar graphs). For example, participants A, B, and C had similar Disinhibition means and standard deviations despite markedly different patterns of responses across days, such as participant B having large peaks and valleys that appear to co-occur with Negative Affect, a pattern not seen in the others. Also, participant D had means close to zero for all composite scores even though Detachment scores were close to 5 on a couple of days. Finally, participant C had long periods of constant Detachment scores, but this information is lost in the descriptives. These are precisely the characteristics – those typically lost in cross-sectional research or when time series data are analyzed in aggregate – that uSEM captures and reflects in individual-level dynamic personality network maps, revealing the person-oriented processes underlying personality.

**FIGURE 4:**
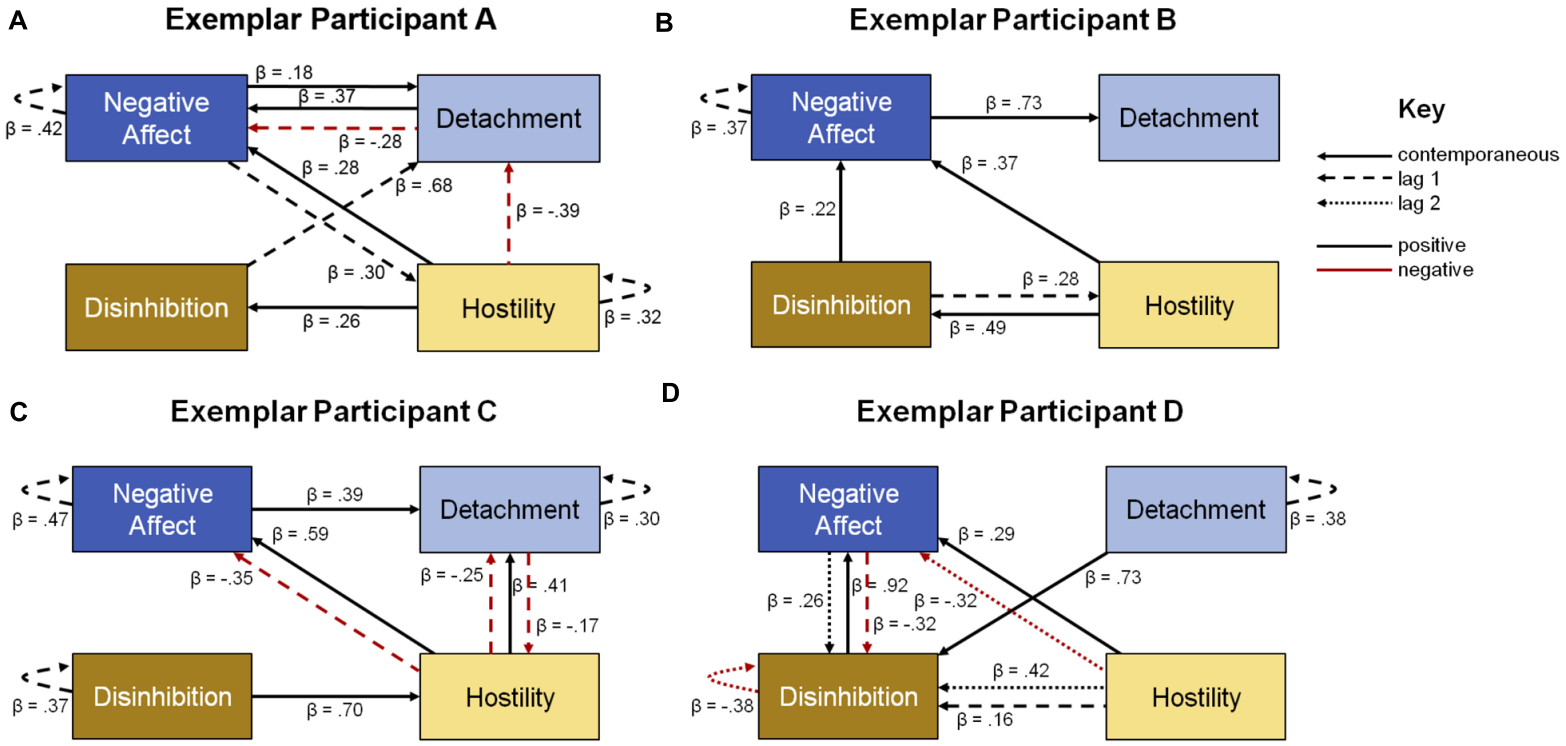
**Network maps from uSEM analyses, showing the interplay among the four within-person personality factors for each of four exemplar participants **(A–D)**.** Solid lines are contemporaneous relations, dashed lines are relations of the first order, dotted lines are relations of the second order, black lines are positive relations, red lines are negative relations, and beta-weights show the magnitude of the relations (all significant at *p* ≤ 0.05). All maps fit the data well; see fit statistics in text.

Model fit can be found in **Table 3**, and the final uSEM maps for each participant are shown in **Figure 4**. The final map for exemplar participant A (**Figure 4A**) fit the data well; multiple solutions were not present, and first order relations were sufficient for capturing all sequential dependencies in the data. The final map for exemplar participant B (**Figure 4B**) fit the data well; four solutions were generated, with the retained solution selected based on lowest AIC and first order relations were sufficient. The final map for exemplar participant C (**Figure 4C**) fit the data well; seven solutions were generated, AIC was again used to select the final solution, and first order relations were sufficient. The final map for exemplar participant D (**Figure 4D**) fit the data well; three solutions were generated, and second order relations were required to capture all sequential dependencies in the data.

**Table 3:** Unified SEM (uSEM) model fit results for four exemplar participants.

	Model fit							AIC for multiple solutions	
Participant	*df*	χ^2^	χ^2^*p*	RMSEA	SRMR	CFI	NNFI	Selected model	Closest alternative
A	12	13.57	0.33	0.025	0.043	0.99	0.98	60.65	N/A
B	16	13.68	0.62	0.000	0.044	1.00	1.00	52.68	53.10
C	12	11.58	0.48	0.000	0.044	1.00	1.00	59.41	60.36
D	28	18.00	0.93	0.000	0.037	1.00	1.00	116.90	129.40

*Models were estimated in LISREL at the lowest temporal order to produce white noise residuals, and multiple solutions were identified during data-driven model fitting. RMSEA, root mean square error of approximation; SRMR, standardized root mean square residual; CFI, Comparative fit index; NNFI, non-normed fit index; AIC, Akaike information criterion.*

The maps can be understood as visual depictions of a series of regression equations (consistent with the beta-weights that accompany the relations), with one equation for each personality factor. A simple example concerns the Detachment of participant B: on any given day, it was positively predicted by Negative Affect occurring on the same day, meaning that increases (decreases) in Negative Affect statistically predicted increases (decreases) in Detachment. This is consistent with the synchronous rise and fall of Negative Affect and Detachment scores visible in the time series plot. A more complex example concerns the Negative Affect of participant A: on any given day, it was explained by Negative Affect (i.e., itself) levels from the previous day, and inversely by Detachment levels from the previous day. Participant A’s Negative Affect was also explained by Hostility and Detachment levels on the same day, and it predicted Detachment levels on the same day as well as Hostility levels on the next day. The set of relations between Negative Affect and Detachment (with reciprocal same day relations, and an inverse prediction of Negative Affect by Detachment) and the pair of relations between Negative Affect and Hostility (with Negative Affect predicting Hostility on the next day, but Hostility predicting Negative Affect on the same day) suggest the presence of feed-forward and feedback mechanisms.

The maps reveal several interesting findings. Visual inspection shows different personality dynamics for each of the participants. For example, the map for participant B was the sparsest (i.e., had the fewest relations with 6), and the maps for the other three participants were equally dense (i.e., had 10 relations). This is an especially interesting finding for participant D, who had composite scores close to zero and second order map relations, suggesting the presence of complex personality processes despite low mean levels of endorsed symptomatology. Graph theoretical metrics, such as total degree (i.e., the number of incoming and outgoing relations for a factor), reveal that Negative Affect was the most important factor for participants A and B, Detachment and Hostility were most important for participant C, and Disinhibition was most important for participant D. This is intuitive in some cases (e.g., Negative Affect also had the highest mean for participant A), but not in others (e.g., Detachment had long periods of constant scores and Hostility had the lowest mean for participant C).

## Discussion

The overarching aim of our study was to provide a conceptual and analytic integration of individual differences research on the structure of psychopathology liability and the complex dynamic processes that comprise mental disorders. First, using data from a sample of individuals diagnosed with PDs who completed several months of (*Mdn* observation *N* = 94) daily diary studies, we tested the BP- and WP-Structures of 16 behaviors chosen to index the broader Internalizing and Externalizing spectra with MSEM (see **Figure 1**). Results indicated that for the BP-Structure, a two-factor model (Internalizing and Externalizing) was sufficient for explaining individual differences in the endorsement of the 16 behaviors, whereas for the WP-Structure a more differentiated four-factor model was supported (Negative Affect, Detachment, Disinhibition, and Hostility). We then demonstrated that the BP-Structure of this model showed specific associations with similar factors derived from traditional psychiatric interviews. Second, using the results of the MSEM, which served to greatly reduce the multivariate modeling space from the individual items to these four factors, we fit a set of idiographic uSEM models to a subset of the participants (*n* = 4) in order to showcase the heterogeneity in dynamic associations among the four daily constructs (i.e., PS-Structures). Thus, our approach represents a hybrid of using MSEM for data reduction and theoretical model testing, followed by the data driven exploration of fine-grained person-specific dynamic processes. We consider each analytic approach and set of results in turn.

### Integrating Structure and Process: Daily Internalizing and Externalizing Behaviors

This study was motivated, in large part, by a tension that has developed in the science of psychopathology; namely, how can models that seek to establish crosscutting dimensions of functioning be reconciled with data collection and analytic approaches that seek to study nuanced contextualized processes? As noted in the introduction, this is a basic tension that has long existed in the personality literature (e.g., Read et al., 2010), which only lately has been given serious theoretical attention (e.g., Fleeson and Jayawickreme, 2015). Adopting some of the conceptual strategies from basic personality science, we tested whether the structure of daily fluctuations in maladaptive behavior conformed to a similar structure derived from individual differences in lifetime psychiatric diagnosis; specifically, the Internalizing and Externalizing spectra. We found that a variant of the hypothesized structure provided a good fit to the data. At the between-person level the items mapped onto a clear two-Factor Internalizing-Externalizing structure. However, the correlation among these two factors was high (*r* = 0.77), indicating that those individuals who report higher average levels of daily Internalizing behavior also report higher average levels of daily Externalizing behavior. Prior research has, in fact, found correlations among lifetime variants of these two factors ranging from modest to strong (range of *r*s = 0.17–0.56) (e.g., Krueger, 1999; Markon, 2010; Røysamb et al., 2011; Forbush and Watson, 2013; Kotov et al., 2011; Wright et al., 2013). Although our final model would suggest these two domains are significantly discriminable, their covariation argues for the importance of considering general severity in daily psychopathology (cf. Kessler et al., 2005; Caspi et al., 2014). Due to the novelty of these analyses, direct comparisons with additional samples are not possible. As such it remains unclear why the covariation between these two factors here is higher than in traditional individual differences work. It may be due to truly higher overlap among daily behaviors, the manner in which MSEM partials between-person variance, the estimator (i.e., robust WLS), the severe nature of the sample, or other factors. Future research will be needed to clarify the degree of overlap among individual differences in these domains derived at the daily level.

More central to our aim, we found that the WP-Structure of daily maladaptive behaviors was more differentiated than the BP-Structure. The identical two-factor Internalizing-Externalizing structure as the between-person model provided good fit to the data by most indices, even as the four-factor structure provided significantly improved fit. The fanning out of content at the four-factor model is consistent with structural models of psychopathology (Markon, 2010; Røysamb et al., 2011; Wright and Simms, 2015) and maladaptive personality traits (Markon et al., 2005; Kushner et al., 2011; Wright et al., 2012; Wright and Simms, 2014) and can be understood as more circumscribed variants of the “pathological Big-4” (Livesley et al., 1998; Widiger and Simonsen, 2005; Calabrese et al., 2012). As such, the resultant 2- and 4-factor structures reflect the hierarchical organization of personality and psychopathology.

It is difficult to overstate the importance of understanding these dimensions as hierarchically organized. In the contem porary era of studying contextualized processes, as researchers seek to study putatively highly specific dynamic phenomena, there will be a pressing need to organize the results of individual studies, highlighting near-neighbor processes for investigation and mapping out “dynamic nomological nets” (cf. Cronbach and Meehl, 1955). As specific dynamic processes are proposed and tested, it will behoove researchers to test for convergent and discriminant validity in near neighbor constructs. For instance, hypotheses that specify processes associated with negative affect should demonstrate discriminability between specific affects and/or detachment related processes. This is a basic approach adopted in individual differences research, and will serve to further clarify specificity and generality in dynamic processes in psychopathological research.

### Estimating and Interpreting Individual uSEM Models and Treatment Implications

Armed with the reduced dimensionality of the four-factor WP-Structure, we then sought to demonstrate that it provides a strong platform for studying dynamic PS-Structures as they play out across days. We approached this by estimating uSEM models at the individual level for four exemplar participants; we mapped the lagged and contemporaneous interplay among Negative Affect, Detachment, Disinhibition, and Hostility for each person. The heterogeneity among these participants is evident in their time series plots (**Figure 3**) and in their network maps (**Figure 4**). The time series showed that each individual tracked different Negative Affect, Detachment, Disinhibition, and Hostility trajectories. For example, participants A and D had Detachment ratings that were mostly low and punctuated by relatively few extreme spikes, whereas participants B and C had Detachment ratings that were constantly changing. The maps showed a highly distinct network of associations among the domains of pathology for each individual. For example, the relations between Negative Affect and Detachment differed among the participants, with Negative Affect positively predicting Detachment for participants B and C, reciprocal positive contemporaneous relations between Detachment and Negative Affect on the same day and Detachment inversely predicting Negative Affect on the next day (perhaps evidencing feed forward and feedback loops) for participant A, and no association between the behaviors for participant D.

In the context of a research study, each of these network maps catalyzes the imagination, leading to questions about how these processes play out in participants’ daily lives. However, in different contexts, specifically clinical settings, one could envision collecting similar data, and using these models to develop hypotheses about a patient’s particular sequence of maladaptive behavior and points of intervention. Take, for example, the uSEM model for participant A. The model suggests that Negative Affect is the lynchpin in this individual’s pathology. Negative Affect, although central to the person’s structure, is more often than not an outcome. Thus, an initial point of intervention may be to address predictors of this individual’s Negative Affect, such as Detachment and Hostility. Hostility, for example, appears to drive same day Negative Affect and Disinhibition, which leads to decreases in next day Detachment, perhaps suggesting pursuing rapprochement with embattled others. This may signal a relative interpersonal strength or healthy functioning that might be leveraged in a treatment. Many additional distinct hypotheses flow from examining the remaining paths across the four maps.

Clinically, the goal would be to disrupt these processes in order to effect change. Yet it would not require distal armchair speculation, as the clinician and patient would have proximal experience with which to augment these quantitative findings. A practitioner could use similar diagrams to those presented here as a tool to engage the patient in a collaborative discussion of how he or she understood his or her own processes, and together develop a target and plan for intervention. This approach of developing hypotheses based on coefficients derived from intensively sampled data and integrating the patient’s own phenomenology is likely viable, as similar methods have been furthered and tested based on traditional dispositional measures (e.g., Finn, 2007). Thus, these results have the potential for direct clinical applicability, at least as a novel tool that can be taken from bench to bedside. In fact, several of the modeling challenges (e.g., multiple well fitting solutions; see Beltz and Molenaar, in revision) may be seen as a boon because they can be presented as alternative hypotheses for the patient to choose from, thereby engaging him or her in his or her treatment. The major rate-limiting step is the development and dissemination of powerful but user-friendly data collection tools, analysis software, and research on the use in clinical practice.

### Selecting an Appropriate Modeling Framework and Alternative Approaches

Refocusing our lens on the methods, we note that there are always a number of decision points to navigate when doing any statistical modeling. With highly multivariate, intensive, longitudinal data across many individuals, the number of possibilities for different analytic approaches is, to say the least, quite large. There were several major considerations that we grappled with, of which we mention two here: (1) adopting a confirmatory vs. exploratory framework, and (2) deciding whether to estimate structures with parameters that varied or were shared across individuals.

First, both confirmatory and exploratory models can be estimated in a MSEM framework. Our primary goal here was to test the degree to which an established model could be fit to a distinct data type. However, different modeling scenarios may compel an exploratory framework. Much needed is basic psychometric and scale development work for item banks to be used in intensive longitudinal data. The same degree of care that has been put into cross-sectional measures has generally not been incorporated in the measures used in dynamic processes (for an exception see Tomko et al., 2014).

Second, one of the exciting possibilities afforded by intensive longitudinal data of the type we modeled here, is that it allows for the estimation of not only the structure of individual differences, but individual differences in structure (i.e., idiographic structures). A challenge for covariance-based idiographic modeling approaches such as uSEM is that they require a minimum of variance in each observed variable in order to be included in the estimation (cf. Nesselroade et al., 2007). In prior studies, which have used many fewer participants, large portions of items have had to be discarded due to lack of endorsement (Nesselroade et al., 2007). This becomes particularly problematic given the use of maladaptive items, which tend to have lower endorsement, even among clinical samples. Even considering the 16 items used here, for many of the individuals specific items would have to be discarded. Therefore, by using MSEM (which estimates the WP-Structure pooled across individuals) or by creating behavioral composites for idiographic analyses, all participants and items can be included in the model.

A related consideration is the optimal degree of complexity for the estimated networks of dynamic processes. For some applications, very specific behaviors may be desired (e.g., in the study of suicidal attempts), but as granularity increases, so too does the potential network complexity. To make this concrete, consider assessing 20 specific negative affect items at each assessment, and analyzing a network of associations among the individual items. This would result in up to 380 possible contemporaneous associations, not considering lagged associations. This would strain direct interpretability, and place limits on the amount of other domains (e.g., social behavior, cognition, motivation) that could be modeled congruently. Naturally, graph theory indices (e.g., node centrality) can be used to winnow down such a highly parameterized model. Alternatively, selecting fewer but broader domains offers desirable qualities like enhanced reliability of assessment, greater bandwidth of measurement, and easier interpretability. The point is that researchers need to be mindful of the optimal level of granularity for their questions of interest.

### Limitations and Future Directions

Several limitations with the current study bear mention. For one, our model derives from the specific set of daily behaviors we chose to measure, and it does not include items related to daily substance use. This is a potential limitation seeing as substance abuse forms a major component of the traditional dispositionally estimated Externalizing domain. Nevertheless, substance abuse is thought to reflect specific instantiations of broader constructs such as Disinhibition or impulsivity, which were well covered in our daily diary data. This is evident in the significant regression path between our interview and daily diary based Externalizing factors. Additionally, the results must be interpreted in the context of this specific sample, which was not a random section of the population, but rather selected to possess elevated psychopathology. Specifically, the current sample was selected for a diagnosis of any PD. Although this ensured breadth of psychiatric diagnoses due to well-established comorbidity patterns, and participants additionally met the criteria for several other clinical syndromes (e.g., anxiety disorders, mood disorders, substance use disorders), future work would benefit from a broader range of severity.

Furthermore, we only estimated and presented uSEM models for four participants. As noted above, this was primarily to demonstrate that, despite strong covariation among factors in the WP-Structural model, individuals exhibit rich and interesting heterogeneity in the dynamic processes constituting that model; the factors within the WP-Structure have contemporaneous and lagged associations with each other that are directional and unique to each participant. Future work should examine the full sample, ideally with a method that can establish shared and unique pathways across individuals (e.g., GIMME; Gates and Molenaar, 2012).

A related issue is that these models were estimated as context independent, and future work is needed that incorporates external variables (e.g., daily stress; cf. Gates et al., 2011). In the current sample we additionally measured a variety of daily stressors, perceived stress in response, several indicators of daily functioning (e.g., sleep, job attendance), and a number of basic behaviors (e.g., affect, social behavior) in the daily diary. In subsequent investigations we plan to examine daily stressors as a contextual input to the system predicting daily fluctuation in psychopathology domains. Similarly, we hope to examine the effect of fluctuations in daily psychopathology on daily functioning. Finally, we hope to test whether a variety of baseline dispositional assessments serve amplify or dampen these within-person linkages in context (stress), psychopathology, and functioning.

### An Agenda for Integrating Empirical Structure and Dynamic Processes in Psychopathology

As this study is the first to attempt to bridge the contemporary empirical thrusts of structural and dynamic investigations into psychopathology, we have merely scratched the surface of what is possible. Here we outline several necessary steps toward more fully realizing the potential of an integrative science of psychopathology.

First, as mentioned briefly above, we must stress the need for measurement development and normative data collection. The items used here were developed ad hoc for this current project as no inventory for intensive repeated measurement of psychopathology (e.g., momentary, daily, etc.) was available. That these items performed extremely well as intended is very encouraging, but that should not preclude more extensive measurement development and refinement. Structural models of individual differences, which have arguably established broad domains of relevant phenotypic functioning (Harkness et al., 2014), provide a firm base from which to launch these measure development excursions. Moreover, there are many other variables that could imbue this work with more psychological texture and nuance. For instance, incorporating motivations and goals in addition to behavior and affect would likely prove fruitful. Going hand in hand with this effort should be the collection of normative data. If intensive repeated measurement is to be used clinically, then established norms, in both the population and treatment samples will be necessary. This will involve more than just importing and applying traditional psychometrics (e.g., means), but the thoughtful application of existing and development of novel “dynamic psychometrics.” At the most basic level this might include relevant measures of variability and instability of behavior over time (e.g., Jahng et al., 2008; Houben et al., 2015), but should conceivably be expanded to include normative associations between daily behaviors, behaviors and environmental antecedents (e.g., What is the average strength of association between daily stress and hostility? Or, between social anhedonia and withdrawal?). Integration of graph theory metrics into the normative data description may also prove fruitful, especially as variable sets increase in number and complexity.

Second, we have focused here on the individual as a closed system, considering neither environmental inputs nor impact on external variables (e.g., other people). However, it is well understood that humans are not closed systems, and indeed as reviewed briefly above contemporary theories of psychopathology are largely based on models of the individual acting in context. Thus, further research in this vein should incorporate traditional inputs and outputs to the system in the form of putative environmental antecedents, stressors, protective factors, and functional concomitants of maladaptive functioning. At the same time we underscore that although traditional perspectives might draw distinctions between environmental and individual located variables (e.g., Cohen et al., 1995) in practice these distinctions are difficult to make, and the current diagnostic nomenclature blends contextual, behavioral, and functional variables. In this regard, the focus on and use of more fine-grained data sampling and analytic approaches may help disentangle the problematically heterogeneous disorder based models of psychopathology.

From this follows our third suggestion, that further work in this area should move forward unencumbered by traditional diagnostic categories. Current models of psychopathology reflect top–down organizational schemes, and are largely studied as such. In the current study we have similarly adopted a top-down perspective in part, in that we used results from quantitative structural models of psychopathology as the starting point for establishing structural models of daily behavior. We believe this provides useful if not necessary scaffolding for the subsequent person-specific analyses. Yet whether one starts with a refined (as we suggest) or an unconstrained set of variables, starting from the bottom–up and seeking out individual differences in dynamic patterns of behavior is an avenue ripe for exploration; especially if combined with techniques that can establish relatively homogenous groupings of individuals based on shared parameters. Building on this, there is a need for more research that takes intensive repeated measurement as a starting point, and seeks to establish functional outcomes that are strongly if not uniquely predicted by resulting parameters (e.g., Stepp et al., 2011; Forbes et al., 2012).

Finally, we believe that this line of research is ideally suited to refining the way in which clinicians assess, diagnose, and monitor treatment effects. For one, the approach is rooted in a dimensional architecture that recognizes that psychopathology varies along gradients of severity, and does not adhere to convenient but arbitrary boundaries. But key is that it incorporates that psychopathology is a process, and therefore should be assessed as such. Thus beyond goals of reducing overall symptom levels, many of the processes hypothesized to drive change in psychotherapy involve not only the decrease in the level of one variable, but also the dynamic change in the association among multiple variables. Indeed, as we have been arguing, psychological symptoms rarely occur in isolation, and instead are coupled with specific contingencies, linked with problematic behaviors, and connected with maladaptive processes. As a result, clinicians often seek not just to decrease a problematic behavior, but also to change the connection between two or more behaviors in order to disrupt the maladaptive processes that maintain psychopathology. Examples include increasing emotional differentiation (i.e., unlink distinct negative emotions), diminishing the link between negative emotions and maladaptive self-regulation (e.g., cutting, substance use, withdrawal), increase positive coping behaviors when distressed, increase tolerance of anxiety in feared situations, and attenuating the link between triggering stimuli and phobic responses. Quantitatively, each of these would be represented by a *dynamic relationship* among variables—or, stated otherwise, an *association that changes over time.* Accordingly, the targeted variables should be assessed in a manner that allows for establishing the strength of these links, and then repeatedly assessed in a way that allows for the continued probing of the strength of that link via appropriate quantitative methods (Wright et al., 2014).

The avenues for future work in this area are wide open, and we have outlined but a few potential directions for a program of research that seeks to integrate structural and dynamic processes. Importantly, much of the fundamental work has yet to be done, starting with measurement, establishing structural similarities, refining the psychometrics, and then moving from a well-established beachhead into more complex and nuanced investigations.

## Conclusion

In sum, we believe the findings presented here are an initial step down one possible path toward merging two contemporary paradigms in psychopathology research, the psychometric approach to establishing crosscutting domains, and the investigation of contextualized dynamic processes. It is our hope that the clearly interpretable factor solutions estimated at the between- and within-person levels demonstrate that domains derived from the study of individual differences in psychopathology can be fruitfully applied and used to organize investigation into person-specific dynamic processes. This approach is already being implemented in basic and applied personality science to establish contingencies and mechanisms driving behavior (e.g., Beckmann et al., 2010; McCabe and Fleeson, 2012), and similar approaches should be viable in psychopathology research.

## Conflict of Interest Statement

The authors declare that the research was conducted in the absence of any commercial or financial relationships that could be construed as a potential conflict of interest.

## Footnotes

1 We examined basic demographics of gender and age, severity of personality disorder based on clinical interview, and average reported daily diary domains that we examined in the study. We found modest correlations of rate of participation with age (*r* = 0.20, *p* = 0.04) but not gender (*r* = 0.17, *p* = 0.10), no association with any of the personality disorders (all *p*’s > 0.10), and modest associations with average daily hostility (*r* = -0.24, *p* = 0.02) and disinhibition (*r* = -0.23, *p* = 0.02) but not the remaining two daily domains (*p*’s > 0.07). Thus, individuals who were younger and reported higher levels of daily Externalizing behavior participated less. However, we believe this had little influence on the results given that (a) these associations are in the context of very high rates of participation (*Mdn* = 94 days; *M* = 89.5 days) and (b) multilevel SEM weights participants contribution to the covariance matrices based on the number of observations.

2 To be comprehensive, we estimated three-factor models at each level, which had a negligible impact on fit at the between-person level, but resulted in significantly poorer fit relative to the four-factor model at the within-person level. We additionally note that changing the estimator to robust maximum likelihood (MLR) resulted in identical conclusions, as did mean and variance adjusted weighted least squares (WLSMV) estimation. In fact, all parameter estimates were identical using the WLSMV estimator, and model fit was substantially improved, but direct comparison of fit cannot be accommodated in a MSEM framework in MPlus. Thus, our results and conclusions are robust to estimation approach.
