# Supplementary material for: Examining the Dynamic Structure of Daily Internalizing and Externalizing Behavior at Multiple Levels of Analysis
Source: Front Psychol. 2015 Dec 17;6:1914. doi: 10.3389/fpsyg.2015.01914 (PMC4681806; doi:10.3389/fpsyg.2015.01914)
Supplement: Supplementary file 1 [file Data_Sheet_1.DOCX]

**APPENDIX: SAMPLE SYNTAX**

**Appendix A:**

**Mplus Syntax for Multilevel Structural Equation Model – Final Model with Interview Factors included**

DATA: FILE = dd100winitialassess.csv;

VARIABLE:

NAMES = id Lability Anxiety Depress Abandon

Apathy Disengage Empty

Anger Offense Aggress Hostile

NegUrg Urg Impuls SenSeek Irrespons

MDDsx DYSsx SOCsx PTSDsx GADsx Antisocialsx Alcoholsx Drugsx

Conductsx Narcissisticsx Borderlinesx

Avoidantsx Paranoidsx Histrionisx Dependentsx;

MISSING ARE ALL(9999);

CATEGORICAL ARE Lability Anxiety Depress Abandon **Indicate categorical variables.**

Apathy Disengage Empty

Anger Offense Aggress Hostile

NegUrg Urg Impuls SenSeek Irrespons;

CLUSTER = ID; **Indicate clustering variable.**

BETWEEN = MDDsx DYSsx SOCsx PTSDsx GADsx **Declare level 2 (i.e., between-person) variables.**

Antisocialsx Alcoholsx Drugsx **Level 1 (i.e., within-person) variables should not**

Conductsx Narcissisticsx Borderlinesx **be declared because you want Mplus to perform a**

Avoidantsx Paranoidsx Histrionisx Dependentsx; **latent decomposition of variance.**

ANALYSIS:

TYPE = TWOLEVEL; **Indicate that this is a two-level analysis.**

ESTIMATOR = WLSM; **Indicate that WLSM estimator is to be used, default is MLR, but with categorical**

**variables and multiple latent factors this is currently computationally prohibitive.**

MODEL:

%WITHIN% **Define 4-factor within-person model.**

NA_W BY Lability Anxiety Depress Abandon;

DET_W BY Apathy Disengage Empty;

HOST_W BY Anger Offense Aggress Hostile;

IMP_W BY NegUrg Urg Impuls SenSeek Irrespons;

%BETWEEN% **Define 2 between-person daily factors and 2 interview factor between-person model.**

INT_B BY Lability Anxiety Depress Abandon

Apathy Disengage Empty;

EXT_B BY Anger Offense Aggress Hostile

NegUrg Urg Impuls SenSeek Irrespons;

INT_I BY MDDsx dysSX DYSsx SOCsx PTSDsx GADsx Avoidantsx Dependentsx Narcissisticsx Borderlinesx;

EXT_I BY Antisocialsx Conductsx Alcoholsx Drugsx Paranoidsx Histrionisx Borderlinesx;

INT_B ON INT_I EXT_I; **Regress daily factors on interview factors.**

EXT_B ON EXT_I INT_I;

OUTPUT: STDYX; **Request standardized results.**

**Appendix B:**

**LISREL Syntax for Unified Structural Equation Model - Participant A**

da no=60 ni=8 ma=km **60 time points for 8 variables (4 factors at t and t-1)**

cm sy fi=covA.txt **Analysis conducted on the block Toeplitz covariance matrix**

mo ny=8 ne=8 ly=id te=ze ps=sy,fi be=fu,fi

le

NA1 DET1 DIS1 HOST1

NA2 DET2 DIS2 HOST2 /

pa be **Analysis begins with empty A (southeast corner) and Φ_1_ (southwest corner) matrices**

0 0 0 0 0 0 0 0

0 0 0 0 0 0 0 0

0 0 0 0 0 0 0 0

0 0 0 0 0 0 0 0

0 0 0 0 0 0 0 0

0 0 0 0 0 0 0 0

0 0 0 0 0 0 0 0

0 0 0 0 0 0 0 0

pa ps **Diagonal covariance matrix of the error process**

1

1 1

1 1 1

1 1 1 1

0 0 0 0 1

0 0 0 0 0 1

0 0 0 0 0 0 1

0 0 0 0 0 0 0 1

nf be(1,1) be(1,2) be(1,3) be(1,4) **Parameters that should not be freed.**

nf be(1,5) be(1,6) be(1,7) be(1,8)

nf be(2,1) be(2,2) be(2,3) be(2,4)

nf be(2,5) be(2,6) be(2,7) be(2,8)

nf be(3,1) be(3,2) be(3,3) be(3,4)

nf be(3,5) be(3,6) be(3,7) be(3,8)

nf be(4,1) be(4,2) be(4,3) be(4,4)

nf be(4,5) be(4,6) be(4,7) be(4,8)

nf ps(5,1) ps(5,2) ps(5,3) ps(5,4)

nf ps(6,1) ps(6,2) ps(6,3) ps(6,4)

nf ps(6,5)

nf ps(7,1) ps(7,2) ps(7,3) ps(7,4)

nf ps(7,5) ps(7,6)

nf ps(8,1) ps(8,2) ps(8,3) ps(8,4)

nf ps(8,5) ps(8,6) ps(8,7)

ou am sl=5 **When there is a single solution, the model automatically iterates (i.e., frees parameters in A and Φ_1_) until freeing a parameter no longer significantly improves the model at *p* < .05**
